# Modeling Celiac Disease: From Pathogenesis to Therapy

**DOI:** 10.3390/ijms27219499

**Published:** 2026-10-24

**Authors:** Vera Rotondi Aufiero, Lucia Treppiccione, Diomira Luongo, Giuseppe Mazzarella, Alessandra Camarca

**Affiliations:** 1 Institute of Food Sciences, National Research Council, 83100 Avellino, Italy; vera.rotondiaufiero@cnr.it (V.R.A.); lucia.treppiccione@cnr.it (L.T.); diomira.luongo@cnr.it (D.L.); giuseppe.mazzarella@cnr.it (G.M.); 2 European Laboratory for the Investigation of Food Induced Diseases (ELFID), University “Federico II”, 80131 Napoli, Italy

**Keywords:** celiac disease, gluten-induced pathogenesis, experimental models, translational research

## Abstract

Celiac disease (CeD) is a common gastrointestinal disorder triggered by the ingestion of gluten in genetically predisposed individuals. It is characterized by a complex immune response involving both adaptive and innate immunity, ultimately leading to intestinal mucosal damage and a broad range of extraintestinal manifestations. Although great progress has been made in understanding the pathogenesis of CeD, several questions remain unanswered. Furthermore, the only treatment currently available is a gluten-free diet, and research is actively working to find therapies that can at least complement dietary treatment. Understanding the complexity of the mechanisms underlying CeD has required the development and use of different experimental models, which allowed the investigation of specific pathways and cell populations. In this review, we provide a critical and updated overview of the main in vitro, ex vivo, and in vivo models used to investigate CeD-related pathways, including human cell lines, ex vivo culturing of duodenal biopsies, intestinal organoids and mouse models. For each model, we highlight its specific characteristics, advantages and limitations, as well as its potential applications in translational research.

## 1. Introduction

Celiac disease (CeD) is an immune-mediated enteropathy positioned between food hypersensitivity and autoimmunity, affecting between 0.7–2.9% of general population worldwide [1,2]. It is a complex disorder in which small-intestinal inflammation and tissue damage are driven by an aberrant immune response to dietary gluten proteins from wheat and related prolamins from rye and barley. CeD has a strong genetic component and develops exclusively in individuals carrying the HLA–DQ2 or HLA-DQ8 molecules. The disease is characterized by infiltration of the intestinal epithelium by intraepithelial lymphocytes (IELs), crypt hyperplasia and villous atrophy, together with the presence of serum antibodies against type2 tissue transglutaminase (TG2). CeD can develop at any age, with a large spectrum of symptoms. The only available treatment remains the lifelong gluten-free diet (GFD) defined in the 1950s by W. Dicke, which does not definitively cure the disease. Moreover, GFD is very constraining, and strict adherence is hard to follow. Consequently, a significant number of patients experience incomplete mucosal healing, more likely due to voluntary or involuntary ingestion of small amounts of gluten [2,3,4,5]. Additionally, a small subset of severely affected patients can develop primary or secondary nonresponse to the GFD, leading to refractory celiac disease (RCD) [4,6]. These limitations highlight the need for new therapies to replace or, at least, to assist GFD.

CeD pathophysiology is multifactorial, requiring the co-existence of several factors and involving different cell types, as described in detail in several excellent reviews [7,8,9,10]. Briefly, gluten is only partially digested by gastrointestinal proteases due to its chemical structure and composition. Gluten-resistant peptides manage to cross the intestinal epithelium and are deamidated by TG2, which enhances their binding affinity to HLA-DQ2/DQ8 molecules. In the lamina propria, the HLA-gluten peptides complexes presented by specialized antigen-presenting cells (APC) activate CD4^+^ T cells, which produce proinflammatory factors, including IFN-γ and IL-21, and interact with B cells, supporting the production of antibodies against gliadin, deamidated gliadin, and the autoantigen TG2. Despite the proinflammatory cytokines produced by these cells boost an inflammatory cascade, stimulating the production of TNF-α and matrix metalloproteases by macrophages and fibroblasts, gluten-specific CD4^+^ T cells are necessary but not sufficient to induce mucosal damage. It has also been demonstrated that gluten may directly stimulate intestinal epithelial cells, inducing the expression of high levels of stress molecules (MICA/B, HLA-E) on their surface, and the secretion of IL-15, a hallmark of CeD mucosa. IL-15 is also produced by myeloid cells in response to gliadin and to other wheat-derived amylase–trypsin inhibitors (ATIs) and plays a central role in CeD pathogenesis. Elevated IL-15 levels promote the activation and cytotoxic reprogramming of IELs, particularly CD8^+^ T cells, leading to epithelial cell targeting and tissue damage.

Despite considerable advances, several aspects of CeD pathogenesis still need to be clarified. These include the roles of non-HLA genes, other triggering factors besides gluten, the possible impact of intestinal microbiota and those of immunometabolism. In addition, it is not well understood how gluten peptides cross the intestinal epithelium, as well as the APC population involved in their processing and presentation. Another big issue concerns how and why oral tolerance is broken, a process directly related to the role of regulatory T cells. Furthermore, the role of γ/δ TCR^+^ cells, which remain elevated also during a GFD, and of other lymphocyte subsets, such as innate lymphoid cells (ILCs), are still under debate. Finally, the early events and the chronological order of pathway activation remain poorly understood, as well as the exact interplay between adaptive and innate immunity and the mechanisms through which the immune response leads to tissue architectural changes.

A deeper understanding of the pathogenetic mechanisms is necessary to improve the chances for a definitive therapy. In fact, the main pathogenetic pathways identified so far, are also targets of several therapeutic approaches currently under development, some of which are being evaluated in clinical trials [11,12,13]. A first line of strategies aims to the reduction of gluten antigenic load reaching the lamina propria, through the employment of specific gluten-degrading enzymes (glutenases), the development of genetically modified wheat, the administration of agents sequestering gluten in the intestinal lumen, or the modulation of tight-junction function. A second line of therapies aims to limit the immune response in different ways: masking immunogenic gluten epitopes through the biotechnological treatment of flours; inhibiting the TG2 enzymatic activity; blocking gliadin presentation to T cells; blocking cytokine signaling; or using enteric-released corticosteroids. Finally, therapies aimed to restore immune tolerance to gluten, based on specific immune-modulating particles able to stimulate regulatory mechanisms, are also under investigation [11,12,13].

A comprehensive dissection of the molecular mechanisms underlying CeD and the evaluation of novel therapeutic strategies remain challenging, mainly because no experimental model can fully reproduce the complex interplay of genetic, immunological, and environmental factors that drive CeD, as well as its diverse clinical manifestations. Furthermore, studies involving celiac patients are often constrained by ethical and technical limitations, highlighting the need for alternative experimental approaches. In this context, in vitro, ex vivo, and in vivo models have been instrumental in advancing our knowledge of CeD pathogenesis. Several complementary experimental systems—including human cell lines, ex vivo organ culture models, intestinal organoids, and animal models, particularly murine models—have been developed and employed to investigate different aspects of the disease. As these experimental systems have evolved, they have progressively shifted the field from a predominantly immune-centered view of CeD toward a more integrated understanding that encompasses tissue architecture, epithelial biology, and cellular interactions within the intestinal microenvironment. Importantly, the existing models are in continuous refinement, progressively enhancing our ability to recapitulate the complexity of CeD, and thereby offering new opportunities for mechanistic studies and the identification of effective therapeutic interventions.

This review provides an overview of the main experimental approaches for studying CeD, with a discussion of specificities, advantages and limitations for each of them. We also consider how the different models have contributed to the advances in CeD knowledge, and to the evaluation of different therapeutic principles that are being developed.

## 2. Cell Models to Investigate CeD-Associated Mechanisms

**Figure 1:**
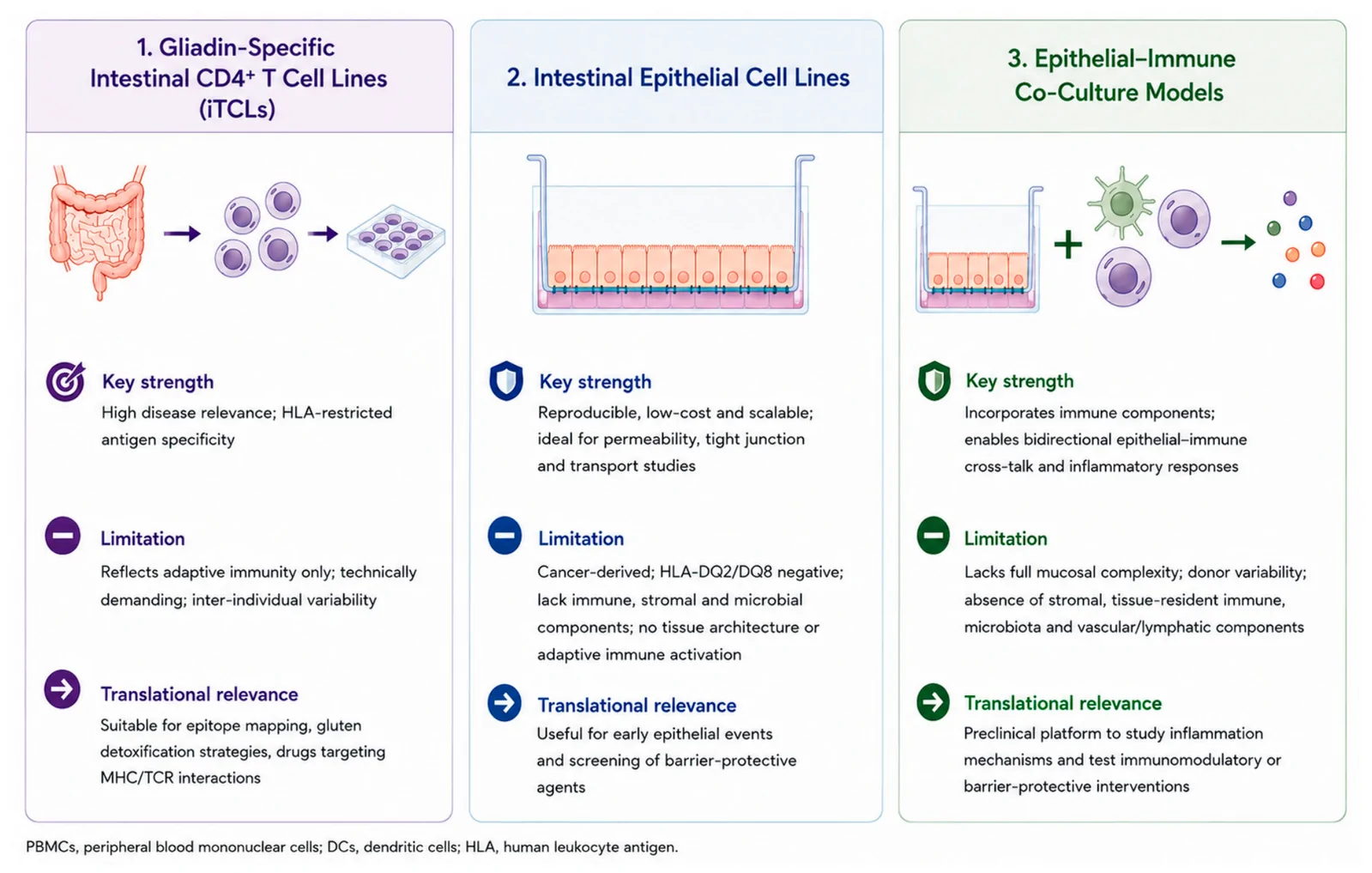
Cell models for celiac disease investigation. Schematic overview of the principal cell-based models employed to investigate the effects of gluten, in vitro. Gliadin-specific intestinal CD4^+^ T-cell lines (iTCLs), derive from duodenal biopsies of CeD patients and enable the study of antigen-specific adaptive immune responses; intestinal tumoral epithelial cell lines (mainly Caco-2) spontaneously differentiate into polarized monolayers with features resembling small-intestinal absorptive enterocytes; co-culturing of epithelial cells with in vitro cultured or ex vivo isolated immune cells, allow to study the interaction between the two cell compartments.

In vitro experimental models have been essential for dissecting the molecular and cellular mechanisms underlying CeD, laying the foundation for understanding the inflammatory mechanisms triggered by gluten. The most widely used in vitro systems include gliadin-specific CD4^+^ T-cell lines expanded from the intestinal mucosa of CeD patients, intestinal epithelial tumor-derived cell lines (mainly Caco-2), and epithelial–immune co-culture models (Figure 1).

### 2.1. Gliadin-Specific Intestinal CD4^+^ T-Cell Lines (iTCLs)

A hallmark of CeD is the presence of gluten-reactive CD4^+^ T cells in the lamina propria, which drive a strong proinflammatory adaptive immune response. These antigen-experienced memory T cells can be expanded ex vivo from intestinal biopsies of CeD patients, generating gliadin-specific polyclonal T-cell lines (intestinal T-cell lines, iTCLs) or individual T-cell clones. iTCLs represent a unique in vitro model that closely reflects disease-relevant HLA-DQ2/DQ8 restricted T-cell responses and enables direct investigation of gluten immunogenicity and T-cell activation pathways.

Early studies demonstrated that gliadin-specific T-cell lines can be generated from CeD patients but not from non-celiac controls [14,15,16]. Subsequent studies showed that gluten-reactive CD4^+^ T cells can also be expanded from patients on a GFD [15,17] and from individuals with positive serology and minimal or absent mucosal lesions (potential CeD) [18,19].

iTCLs are typically generated from small-intestinal biopsies using protocols that differ mainly in the use of collagenase digestion and in the separation of epithelium from lamina propria [18,20]. Further stimulation of intestinal lymphocytes with gliadin, or defined gluten peptides, in the presence of autologous irradiated peripheral blood mononuclear cells (PBMCs) as antigen-presenting cells enhances the specificity and the stability of the iTCLs.

Long-term maintenance is achieved through repeated stimulation cycles using heterologous PBMCs as feeder cells, phytohemagglutinin, and IL-2. Antigen specificity is commonly assessed through T-cell proliferation assays and cytokine readouts, particularly IFN-γ production.

iTCLs have played a central role in defining CeD immunopathogenesis. Early evidence demonstrated that the gluten-specific response is dominated by Th1-like cytokine production [15,16,21], and later studies confirmed the presence of additional gluten-reactive subsets, including Th17 [22] and regulatory populations [23]. In addition, short-term iTCL cultures, analyzed after a limited number of stimulation cycles, have been used to compare immune-cell populations across different Marsh grades, enabling the characterization of disease-stage-associated shifts in mucosal lymphocyte composition (e.g., IL-4^+^ T-cell reduction and increased γδ TCR^+^ cells during progression to Marsh III lesions) [24,25].

iTCLs and T-cell clones have also been fundamental in clarifying the role of TG2-mediated deamidation in enhancing gluten immunogenicity and binding to HLA-DQ2/DQ8 [26,27]. They have enabled the mapping of immunodominant epitopes restricted by HLA-DQ2.5, HLA-DQ2.2, and HLA-DQ8 [20,28,29], providing the experimental foundation for identifying pathogenic gluten sequences and for characterizing gluten-specific TCR repertoires [29,30].

Beyond mechanistic studies, iTCLs represent a key preclinical tool to assess the immunogenic potential of cereal-derived proteins and processed foods. They have been used to evaluate alternative cereals and pseudocereals (e.g., oats, teff, millet, quinoa, amaranth) [31,32] and to test the immunogenicity of gluten in processed products [33,34]. Moreover, they have been widely employed to validate gluten detoxification strategies, including gluten-degrading enzymes [35,36,37,38,39,40] and gluten transamidation approaches based on microbial transglutaminase and acyl-acceptor molecules [41]. These systems have also supported the development of low-immunogenic wheat lines through amino acid substitutions or RNA interference targeting prolamins [42,43,44].

iTCLs have additionally been used to evaluate immunomodulatory strategies aimed at dampening pathogenic T-cell activation, including exposure to IL-10 or mesenchymal stem cells [45,46], as well as treatment with chitosan [47]. Finally, different approaches targeting HLA–peptide complexes have also employed iTCLs. In this context, TCR-like monoclonal antibodies directed against HLA-DQ2.5 loaded with immunodominant gliadin peptides have demonstrated inhibitory activity against antigen-specific T-cell activation [48]. Other approaches evaluated using iTCLs include HLA-DQ2/DQ8 gliadin antagonist peptides [49] and recombinant HLA-DQ2.5/peptide molecules capable of blocking pathogenic CD4^+^ T-cell responses [50].

Overall, the combination of high disease relevance and preserved antigen specificity makes iTCLs a valuable translational tool for advancing our understanding of CeD pathogenesis and for supporting the development and validation of targeted therapeutic interventions. Nevertheless, iTCLs represent a simplified model that primarily reflects the adaptive immune component of CeD and does not fully recapitulate the complexity of the intestinal mucosal microenvironment. In addition, their generation and expansion are technically demanding, requiring access to patient-derived intestinal biopsies and specialized laboratory expertise, while their performance may be influenced by inter-individual variability. Prolonged in vitro culture and repeated antigenic stimulation can also alter the physiological composition of T-cell populations, potentially leading to the preferential expansion of highly proliferative clones and reducing their similarity to in vivo immune responses.

### 2.2. Intestinal Epithelial Cell Lines

Intestinal epithelial dysfunction and barrier impairment are key early events in CeD pathogenesis [51,52]. Human intestinal epithelial tumor-derived cell lines have therefore been widely used to model epithelial responses to gluten-derived peptides. Among them, Caco-2 cells are the most extensively used system.

Although derived from a human colon adenocarcinoma, Caco-2 cells spontaneously differentiate into polarized monolayers with features resembling small-intestinal absorptive enterocytes, including brush-border formation and tight-junction development [53].

Caco-2 cells can be cultured as monolayers or grown on transwell inserts to generate polarized epithelia. This configuration allows independent access to the apical and basolateral compartments and enables quantitative evaluation of barrier integrity through transepithelial electrical resistance (TEER) and paracellular permeability assays. TEER is determined by measuring the electrical resistance across the epithelial monolayer, by applying a small alternating current using a pair of electrodes positioned in the apical and basolateral compartments. The instrument records the voltage drop across the cell layer and converts it into an electrical resistance value that reflects the tight junctions’ integrity. High TEER values indicate well-formed, low permeability junctions, whereas decreases in resistance correspond to junctional opening and increased paracellular flux. This makes Caco-2 cells a convenient model to investigate epithelial permeability, transport mechanisms, and early inflammatory signaling. Cells are typically exposed to gliadin digests or defined gliadin peptides (e.g., p31–43/p31–49), often under conditions that mimic inflammatory stress.

Studies on Caco-2 cells have demonstrated that gliadin peptides can directly induce epithelial stress responses, including rapid decreases in TEER and increased paracellular permeability [54,55]. In addition, gliadin exposure has been associated with oxidative stress responses, increased reactive oxygen species (ROS) generation, mitochondrial dysfunction, and metabolic alterations [56,57,58,59].

Caco-2 cells have also been used to investigate intracellular pathways triggered by gliadin, including calcium-dependent signaling, endoplasmic reticulum (ER) stress, and activation of TG2-dependent pathways [60]. Furthermore, these models have contributed to the characterization of epithelial cytokine and chemokine release, supporting the concept that intestinal epithelial cells actively contribute to inflammation rather than serving merely as passive barrier components [61].

Another major contribution of Caco-2 models has been the analysis of gliadin peptide transport across the epithelial layer. Studies have demonstrated that immunogenic peptides can translocate to the basolateral compartment and that secretory IgA may facilitate epithelial transport mechanisms [62,63]. From a translational perspective, Caco-2 systems have been widely applied to test therapeutic strategies, including probiotics and postbiotics [64,65,66], gluten-degrading enzymes [67,68], neutralizing polymeric gliadin binders [69], antioxidants [59,70], agents aimed at modulating tight-junction integrity such as zonulin pathway inhibitors [71] and even the TG2 inhibitor ZED1227 [72].

Some studies have employed IEC-6 cells, that are intestinal epithelial cell line derived from the crypts of normal rat jejunum. IEC-6 cells are considered non-transformed (non-cancerous) and retain many characteristics of normal intestinal epithelial cells, making them useful for investigating mechanisms involved in intestinal physiology and injury repair. In CeD research, these cells have been used to investigate gluten-induced effects and zonulin release, as well as serving as a substrate in immunofluorescence assays to detect anti-actin autoantibodies (AAA), which are highly specific serological markers of intestinal villous atrophy in patients. They have also been used to test the efficacy of supplements or pharmaceutical agents designed to protect the gut.

More recently, the murine small-intestinal enterocyte cell line Mode-K has been proposed as a complementary epithelial model. Compared with Caco-2, Mode-K cells may better reflect small-intestinal epithelial susceptibility to oxidative and inflammatory damage and have been reported to be more sensitive to gliadin-induced toxicity [73]. This system may therefore represent a useful alternative for investigating epithelial stress responses and redox imbalance in a murine intestinal context.

Epithelial cell line models remain valuable for studying early epithelial events, screening barrier-protective agents, and validating mechanistic pathways that can subsequently be investigated using ex vivo organ culture or animal models. In addition, they provide reproducible, low-cost, and scalable platforms that are also suitable for high-throughput screening. A major limitation is their cancer-derived origin, which may affect differentiation programs, signaling pathways, and immune responsiveness. Furthermore, they are HLA-DQ2/DQ8 negative and lack immune, stromal, and microbial components, thereby reducing their physiological relevance. Finally, these models cannot reproduce tissue architecture, crypt–villus dynamics, or the adaptive immune activation central to CeD pathogenesis.

### 2.3. Epithelial–Immune Co-Culture Models

CeD is driven by a complex interplay between epithelial stress, innate immune activation, and adaptive T-cell responses. To partially reproduce epithelial–immune crosstalk in vitro, several studies have developed co-culture models combining epithelial cells with immune cells derived from PBMCs or myeloid cell lines.

The simplest co-culture systems involve polarized Caco-2 monolayers exposed to gliadin peptides in the presence of PBMCs placed in the basolateral compartment. Variants of this model use purified monocytes or macrophages derived from PBMCs or standardized myeloid cell lines such as THP-1 or U937, reducing donor variability.

More complex models include 3D multicellular systems incorporating epithelial cells, immune cells, and stromal components such as fibroblasts. For example, Truzzi et al. developed a three-component 3D intestinal model including Caco-2 cells, U937 immune cells, and L929 fibroblasts to assess inflammatory responses induced by gliadins and glutenins from different wheat cultivars [74].

Co-culture models have demonstrated that gliadin exposure can induce not only epithelial barrier impairment but also immune activation and cytokine amplification. In particular, co-culture with PBMCs has been used to demonstrate increased secretion of inflammatory cytokines, such as TNF-α and IL-6, supporting the concept that epithelial alterations can synergize with immune activation to amplify inflammation [75,76].

Multicellular models have been applied to compare the proinflammatory potential of ancient versus modern wheat proteins by evaluating endpoints such as IL-8 production, epithelial proliferation, and tight-junction protein expression [74].

In conclusion, co-culture systems represent an intermediate level of complexity between epithelial monocultures and tissue-based ex vivo models, and fill a methodological niche enabling the investigation of bidirectional epithelial–immune signaling, under controlled condition. Nonetheless, PBMC-based systems exhibit high inter-donor variability and are difficult to standardize. Moreover, most co-culture models do not include key stromal compartments, tissue-resident immune cells, microbiota-derived factors, vascularization, or lymphatic components, limiting their ability to fully reproduce mucosal remodeling and chronic inflammation.

## 3. Modeling Intestinal Tissue in Celiac Disease

**Figure 2:**
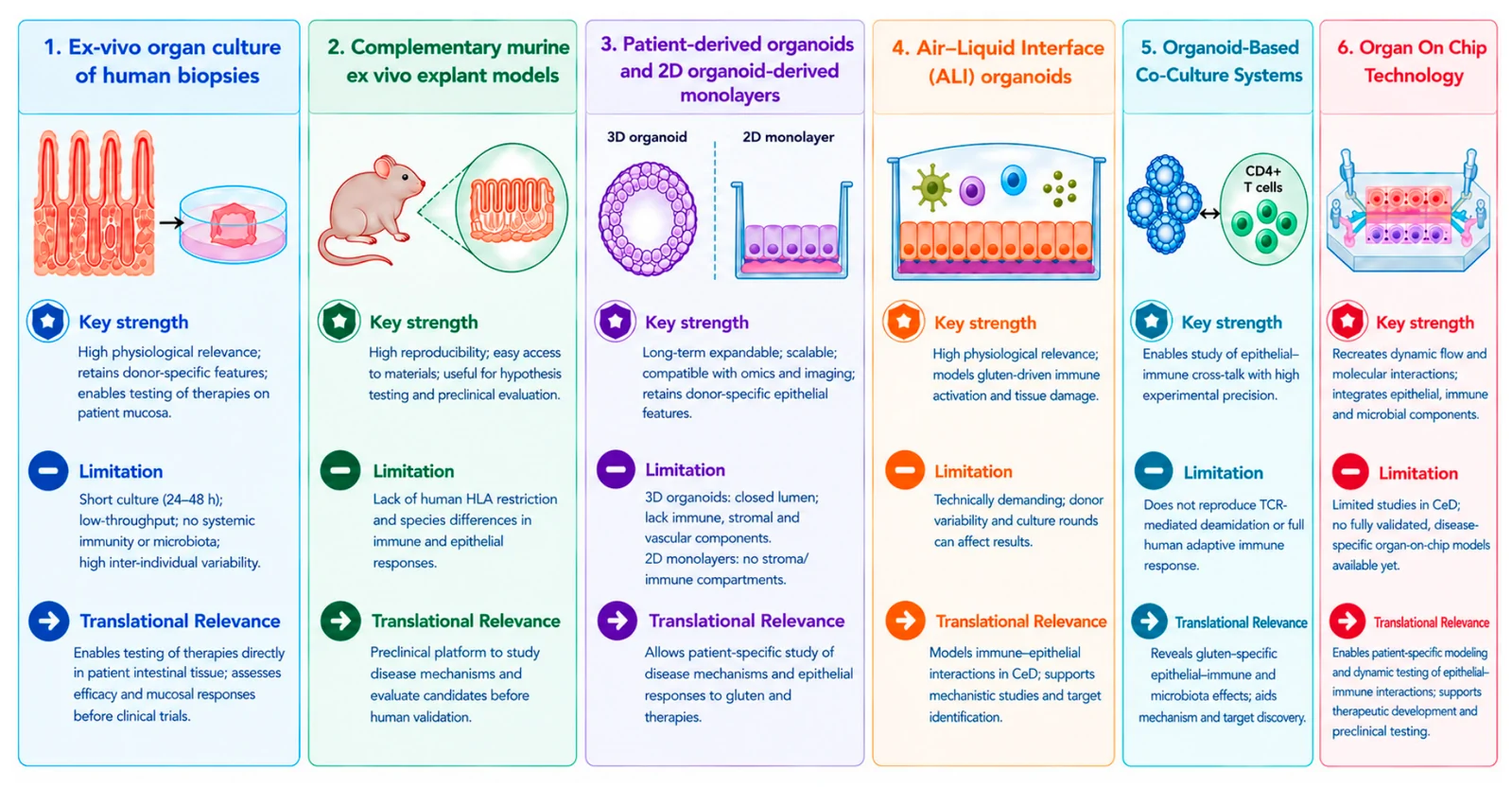
Modeling intestinal tissue in CeD. Human biopsies may be cultured ex vivo using the organ culture system; murine explant model defined GEV (Gut Ex-Vivo System) are based on small-intestinal explants derived from gluten-sensitive mice; patient-derived organoids and organoid-derived monolayers reproduce the patients epithelial compartment; patient-derived air–liquid interface (ALI) organoids include epithelial, stromal, and immune components; organoid-based co-culture systems have been reported from a transgenic mouse; organ-on-chip technologies are also being developed, that can be used for CeD.

The investigation of CeD pathogenesis in vivo is limited by ethical constraints and by the difficulty of monitoring early immunological events directly within the human intestinal mucosa. For this reason, ex vivo intestinal models have become essential experimental tools to study gluten-induced responses under controlled conditions. Compared with conventional in vitro systems, ex vivo approaches provide higher physiological relevance because they preserve key structural and functional features of the intestinal mucosa, including epithelial architecture, differentiation status, and—in selected models—immune and stromal interactions (Figure 2).

### 3.1. Ex Vivo Organ Culture of Human Biopsies

Ex vivo intestinal organ culture relies on the short-term maintenance of small-intestinal biopsy specimens, usually obtained from the duodenal mucosa, under controlled culture conditions that preserve tissue viability, epithelial morphology, and overall mucosal organization.

The model was first developed by Browning and Trier, who demonstrated that human small-intestinal biopsies could be successfully maintained outside the body for 24–48 h while retaining key structural and functional properties, including villous architecture, epithelial differentiation, and metabolic activity [77]. In this system, biopsy samples are generally positioned on mesh or grid supports and cultured at the air–liquid interface in RPMI-based media under controlled temperature and oxygen conditions. The preservation of tissue integrity during this period allows the application of multiple analytical approaches, including histological, immunohistochemical, molecular, and biochemical assessments.

Early studies using intestinal organ culture demonstrated that exposure of duodenal biopsies from patients with CeD to gliadin proteins induces rapid mucosal injury, including epithelial disruption and villous shortening, within 24–48 h [78,79]. Subsequent refinement established that short-term gliadin stimulation can reproducibly induce key histological and immunological hallmarks of CeD, including villous blunting and increased intraepithelial lymphocytes, while maintaining the native tissue organization [57].

Importantly, multicenter studies demonstrated that organ culture combined with immunohistochemical readouts (e.g., CD3^+^ IEL counts) can distinguish patients with CeD from non-celiac controls, including individuals on a GFD, suggesting that this approach may provide a functional alternative to in vivo gluten challenge [80,81], and may be useful for diagnostic purposes in uncertain cases. In addition, this model allows the detection of autoantibodies produced locally within the mucosa, including anti-endomysial antibodies (EMA) and anti-TG2 antibodies, making it particularly valuable for confirming the diagnosis in seronegative or diagnostically ambiguous cases [80,81].

Beyond diagnostic applications, organ culture has been widely used to investigate multiple aspects of CeD pathogenesis, ranging from early studies demonstrating the ability of different gluten and gliadin fractions to induce inflammation and T cells activation [82], to studies dissecting the role of HLA class I-restricted CD8^+^ T cells in inducing enterocyte apoptosis, [83] and others addressing the molecular mechanisms underlying direct, T-cell independent effects of gliadin peptides (specifically p31–49), which may interfere with endocytosis and the EGF pathway [84]. The finding that the same peptide can induce expression of the nonclassical HLA class I molecule MICA in biopsies from patients with active CeD, but not in control biopsies, has also been important [85]. Other studies have focused on the mechanisms involved in the transport of gluten-derived peptides across the intestinal epithelial barrier [86]. Moreover, gliadin fragments have been shown to increase intestinal permeability in both subjects with CeD and non-celiac controls [87] and to activate zonulin signaling irrespective of the genetic predisposition to autoimmunity, thereby increasing intestinal permeability to macromolecules [71]. In addition, this approach has been used to characterize IL-17A-producing cells in the celiac mucosa [88,89] and to investigate the influence of IFN-α-producing dendritic cells and IL-21 on Th1 responses in CeD [90,91].

Organ culture studies have also been instrumental in defining early innate immune activation, demonstrating that gliadin rapidly induces IL-15 expression, promoting epithelial stress responses and activation of IELs [92]. Consistently, Nanayakkara et al. reported that gliadin stimulation triggers a combined epithelial stress and proliferative response, involving IL-15 induction and type I interferon signaling [93]. Other investigations using duodenal biopsy cultures have demonstrated that regulatory T cells are present in the mucosa of untreated patients with CeD and that their suppressive activity is impaired because of increased IL-15 expression [94].

More recently, the effect of an anti-IL-15 monoclonal antibody has been evaluated by this model. Treatment of CeD biopsies with this antibody was shown to reduce intraepithelial lymphocyte cytotoxicity, epithelial apoptosis, and stress markers expression, highlighting the translational potential of this platform for testing targeted therapies [95]. Other therapeutic strategies evaluated using organ culture include the zonulin antagonist AT1001 (larazotide acetate), which was shown to prevent gliadin-induced increases in intestinal permeability, supporting tight-junction modulation as a potential treatment approach [71]. Organ culture models have also facilitated the assessment of enzymatic detoxification strategies. Stenman et al. demonstrated that gliadin pretreated with proteases from germinating wheat induced lower immune activation, as evidenced by reduced CD25^+^ lymphocyte activation and decreased production of CeD-associated autoantibodies. Similarly, Lombardi et al. reported that microbial transglutaminase treatment of gliadin peptides reduced mucosal immune activation [96].

Finally, organ culture is also a valuable tool for investigating both innate and adaptive immune responses triggered by gluten-derived peptides and other proteins from alternative cereals, such as different cultivars of *Triticum monococcum* (*Tm*) [97], oat varieties (*Avena potenza* and *Avena genziana*) [32] and quinoa [98]. The specific effect of ATIs proteins from *Tm* on mucosal immune activation have also been investigated [99].

The main advantage of intestinal organ culture is its high physiological relevance. By preserving villous architecture and mucosal compartmentalization, this model allows evaluation of epithelial injury, immune activation, and cytokine-driven stress pathways within a human tissue context. It retains donor-specific characteristics and therefore enables investigation of inter-individual variability. In addition, the system provides a powerful platform for testing candidate therapies directly on patient-derived mucosa, including barrier-protective compounds and immune-targeting interventions. A major limitation of intestinal organ culture is the availability of biological samples, given the invasive nature of gastroduodenoscopy. In addition, the short culture duration (typically 24–48 h), restricts the investigation of long-term remodeling processes such as crypt hyperplasia and chronic fibrosis. Moreover, tissue viability and responsiveness can vary depending on biopsy quality, oxygenation conditions, and inter-individual differences. Standardization across laboratories remains challenging. Finally, the model cannot reproduce systemic immune priming, lymphoid organ responses, or microbiota-mediated effects occurring in vivo.

### 3.2. Complementary Murine Ex Vivo Explant Models

Murine intestinal explant systems have been explored as an alternative to human biopsy-based models, offering a more controlled, reproducible, and cost-effective experimental setting, while avoiding many of the limitations associated with the availability and variability of human biological samples. One notable example is the Gut-Ex-Vivo System (GEVS) developed by Gagliardi et al., which utilizes small-intestinal explants derived from gluten-sensitive BALB/c mice maintained under gluten-free conditions and subsequently challenged with gliadin. The explants are cultured in a dynamic chamber specifically designed to preserve tissue viability and architecture. Remarkably, this system reproduces within 16 h, many of the biochemical and morphological alterations observed in vivo after four weeks of gliadin exposure. Gliadin exposure resulted in downregulation of the cystic fibrosis transmembrane conductance regulator (CFTR) and concomitant upregulation of TG2. These changes were accompanied by increased intestinal permeability, enhanced production of proinflammatory cytokines, including IL-15, IL-17, and IFN-γ, and significant morphological alterations of the intestinal epithelium and villi [100].

The GEV model has been recently used to investigate the activation of ER stress signaling pathway by gliadin.

The results support a key role for ER stress in CeD pathogenesis and identify chemical chaperones as potentially valuable therapeutic agents [101].

Although murine explants provide higher reproducibility and easier access to experimental material than human biopsies, their translational relevance is limited by the absence of human HLA restriction and by differences in immune and epithelial responses between mice and humans.

### 3.3. Patient-Derived Organoids (PDOs) and 2D Organoid-Derived Monolayers

The organoid system was pioneered by Sato et al., who demonstrated that Lgr5^+^ crypt stem cells can generate a self-renewing intestinal epithelium capable of multilineage differentiation [102,103]. Organoids are self-organized, three-dimensional structures containing organ-specific cell types. Given the affected site, small-intestinal organoids can be used to study CeD. They reproduce key features of intestinal epithelial organization, including crypt-like domains, villus-like differentiation, and generation of multiple epithelial cell lineages. In CeD research, patient-derived organoids (PDOs) are typically established from duodenal biopsies by isolating crypts through mechanical dissociation and chelation (e.g., EDTA), followed by embedding in extracellular matrix scaffolds (e.g., Matrigel) and culturing them in growth factor–supplemented media [104,105,106]. To overcome the limitations of closed-lumen 3D organoids, organoid-derived 2D monolayers have been developed by dissociating organoids and seeding epithelial cells on permeable supports to form polarized monolayers with accessible apical and basolateral surfaces.

While 3D organoids reproduce epithelial heterogeneity and regenerative capacity, 2D monolayers are particularly well-suited to quantitative functional assays of epithelial barrier integrity [106]. These models allow TEER measurements and permeability assays, making them well-adapted for investigating tight-junction dysfunction and epithelial permeability changes relevant to CeD.

Interestingly, PDOs derived from patients CeD exhibit distinct morphological, transcriptional, and functional features compared with those from healthy controls, indicating that disease-associated epithelial alterations can persist independently of ongoing gluten exposure [104,105,106]. These organoids retain donor-specific characteristics and show altered expression of genes involved in stem cell function, epithelial differentiation, extracellular matrix remodeling, and cell adhesion, supporting the concept of an intrinsic epithelial contribution to disease susceptibility [104,105]. Moreover, CeD-derived organoids display persistent inflammatory and stress-related signaling together with impaired barrier function, including reduced expression of ZO-1 and other barrier-associated genes such as CLDN18, MUC5AC, and MUC6, suggesting the presence of a stable disease-associated epithelial “memory” [104,105]. More recently, a bidirectional relationship between impaired autophagy and inflammation has been demonstrated, with CeD organoids exhibiting defective autophagic flux, activation of the mTOR and NF-κB pathways, and increased production of proinflammatory mediators [107]. Reduced expression of the phosphatase PTPRK has also been identified as a key driver of enhanced EGFR/ERK signaling and enterocyte proliferation in CeD, implicating dysregulated epithelial growth pathways in disease pathogenesis [108]. In addition, CeD enterocytes display persistent activation of inflammatory pathways, including NF-κB, ERK, IL-1β, and IL-6, together with heightened responsiveness to gliadin peptides and viral-like stimuli [109].

Together, these studies indicate that CeD organoids retain intrinsic defects in autophagy, proliferation, and inflammatory signaling, supporting their value as models for investigating disease mechanisms and identifying novel therapeutic targets.

From a translational perspective, PDOs have been used, for example, to study the effects of microbiota-derived bioproducts and postbiotics derived from *Lactobacillus rhamnosus* GG on inflammatory pathways triggered by gliadin [104,110]. Moreover, organoid-derived from patients following in vivo administration of the TG2 inhibitor ZED1227 have been used to investigate the effects of drug administration on the epithelial cell transcriptome [111].

A major advantage of organoids is their long-term expandability, scalability, and compatibility with transcriptomic, proteomic, and imaging approaches. They enable the dissection of epithelial-specific pathways without confounding effects from immune cells and can be derived directly from patient tissue, retaining donor-specific genetic and functional features. Nevertheless, classical 3D organoids have a closed lumen, limiting direct access to the apical surface and complicating modeling of luminal gliadin exposure. In addition, epithelial-only organoids lack stromal, immune, vascular, and neural compartments, reducing their physiological complexity and limiting their ability to reproduce immune-mediated epithelial injury typical of active CeD.

### 3.4. Air–Liquid Interface (ALI) Organoids

Air–liquid interface (ALI) organoids represent an important, advanced ex vivo-derived culture system that partially preserves multicellular tissue complexity. Unlike epithelial-only organoids, ALI cultures can maintain epithelial, stromal, and immune components, enabling investigation of immune–epithelial crosstalk in response to gluten stimulation. ALI organoids are established from biopsy fragments embedded in a matrix, with the apical surface exposed to air. In the context of CeD, ALI organoids were first developed by Santos et al. who demonstrated that gliadin induces epithelial injury and cell death, expansion and activation of CD4^+^ and CD8^+^ T cells, and production of anti-TG2 autoantibodies, thus reproducing major hallmarks of active CeD. Based on these characteristics, the authors proposed that ALI organoids could be used for diagnostic assessment in patients with suspected CeD, irrespective of whether they follow a gluten-free or gluten-containing diet. A key mechanistic insight from this study was the identification of IL-7 as a mediator of epithelial damage. Gliadin stimulation induced IL-7 production primarily by mesenchymal cells, which promoted CD8^+^ T-cell cytotoxic activation through the upregulation of NKG2D and NKG2C. Blocking IL-7 signaling reduced epithelial damage, suggesting a novel pathogenic pathway and potential therapeutic target. IL-7 blockade was sufficient to inhibit gliadin-induced villous atrophy, revealing a prominent and previously undescribed role of IL-7 in CeD [112].

ALI organoids represent one of the most advanced ex vivo platforms for studying multicellular mechanisms in CeD. Nevertheless, no additional studies using this experimental approach have yet been reported. Major limitations include the technical demands of these systems and their relatively low throughput. Tissue viability and immune-cell composition may also vary substantially depending on biopsy quality, culture conditions, and successive expansion rounds. For these reasons, standardization of protocols across laboratories is crucial to enable the use of ALI organoids for mechanistic discovery and for testing immune-targeting therapies in a tissue-like setting that reproduces epithelial–immune interactions.

### 3.5. Organoid-Based Co-Culture Systems

As an alternative to patient-derived organoids containing autoimmune disease-associated features (see Section 3.4 on ALI organoids), organoid-based co-culture systems incorporating immune components have been developed, mainly in murine settings. These models aim to reproduce epithelial–immune signaling relevant to IEL activation and cytokine-driven tissue injury, in a mouse model context. Rahmani et al. [113] reported that organoid-derived monolayers from gluten-immunized DR3-DQ2.5-hCD4 mice can express MHC class II and, when co-cultured with splenic gluten-specific CD4^+^ T cells isolated from immunized mice, are able to present cognate antigens to CD4^+^ T cells, inducing proliferation and secretion of cytokines including IL-2, IFN-γ, and IL-15. Interestingly, in this study, organoid monolayers and CD4^+^ T-cell co-cultures were incubated with gluten, that had either been predigested with elastase-producing *Pseudomonas aeruginosa* or left untreated. The results demonstrated that gluten metabolized by *P. aeruginosa*, but not by the *lasB* mutant, enhanced CD4^+^ T-cell proliferation and activation [113]. These systems allow mechanistic investigation of cytokine-driven epithelial–IEL interactions and can be manipulated experimentally with high precision. However, they do not fully reproduce TG2-mediated deamidation processes or the complete human adaptive immune response, limiting direct translational applicability.

### 3.6. Organ-on-Chip Technology

Recent advancements in organ-on-chip technology offer promising opportunities to model the complex intestinal microenvironment of CeD, although their application in this field remain at an early stage. While several gut-on-chip systems have been successfully developed to study intestinal barrier function and host–microbe interactions, only a few efforts have focused specifically on replicating CeD pathogenesis in a chip-based platform. For instance, Moerkens et al. proposed a conceptual “celiac disease-on-chip” model incorporating patient-derived induced pluripotent stem cells (iPSCs), intestinal epithelium, immune cells, and environmental factors such as gluten and microbiota to recapitulate disease-specific features in vitro under dynamic flow conditions [114]. Similarly, recent reviews have highlighted the potential of integrating human intestinal organoids with organ-on-chip platforms to better recapitulate the multifactorial nature of CeD, particularly through the incorporation of immune components, patient-specific features, and dynamic monitoring of epithelial responses [115]. Furthermore, an ongoing research project from the University of Latvia aims to establish a polydimethylsiloxane (PDMS)-free chip-based model of CeD by co-culturing intestinal epithelial cells, endothelial cells, and patient-derived PBMCs exposed to gluten (https://www.cfi.lu.lv/en/modelling-celiac-disease-on-a-chip, accessd on 21 October 2026). This approach is intended to enable the study of barrier integrity, cytokine responses, and immune–epithelial interactions under near-physiological conditions. Despite their promise, fully validated and disease-specific organ-on-chip models for CeD remain scarce.

## 4. In Vivo Mouse Models Used to Investigate of Celiac Disease

Although in vitro and ex vivo systems have provided valuable tools for investigating the specific cellular and molecular mechanisms underlying CeD, they cannot fully reproduce the complexity of the disease. For this reason, several mouse models have been developed over the years to reproduce specific features of CeD. Among these, murine models are the most widely used, owing to their genetic manipulability, cost-effectiveness, and the availability of sophisticated immunological tools. These models are indispensable for studying systemic immune priming, antigen presentation, lymphocyte trafficking, and the long-term consequences of gluten exposure, while also providing valuable platforms for preclinical evaluation of novel therapeutic strategies.

**Figure 3:**
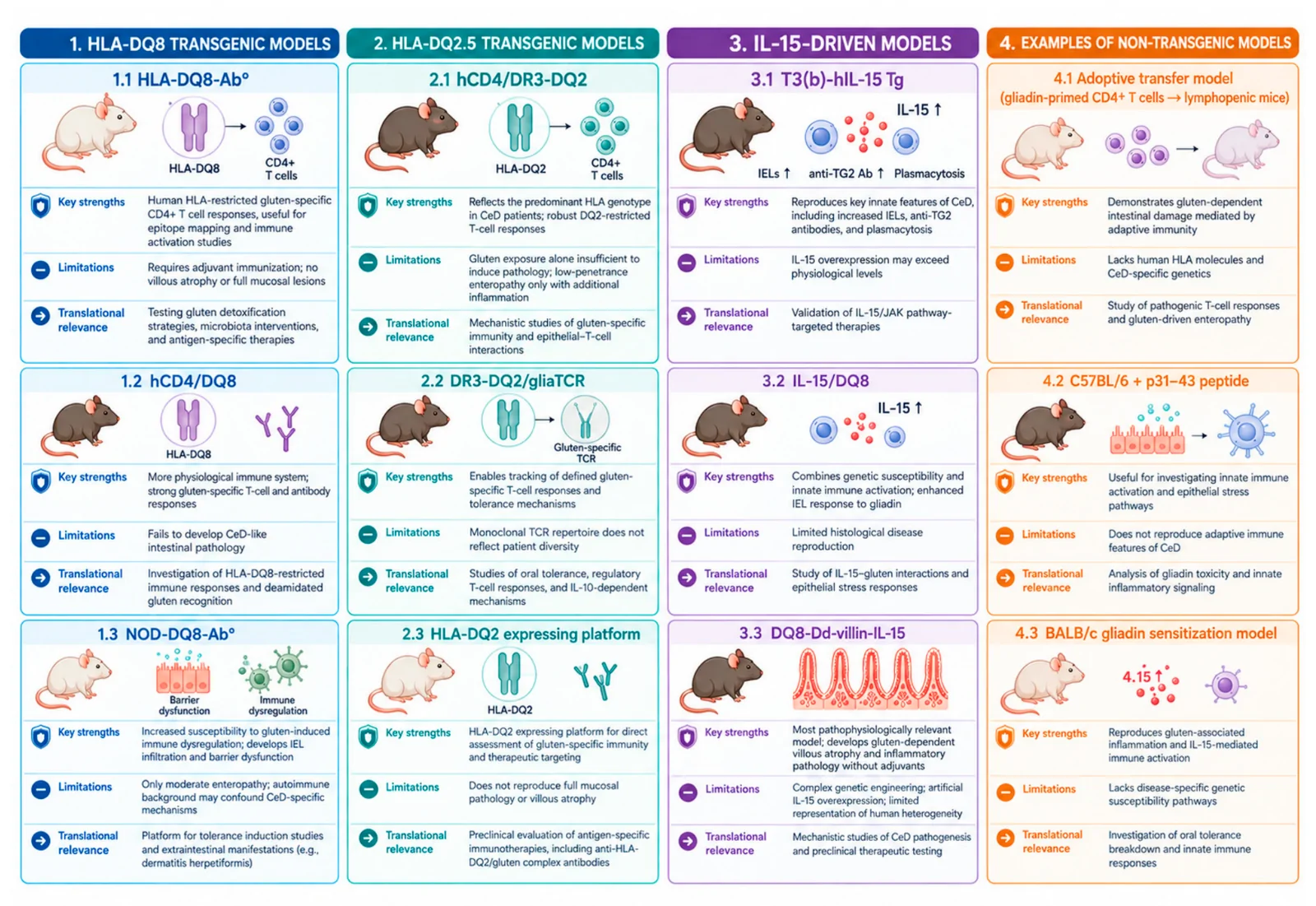
Main mouse models used to investigate celiac disease pathogenesis. Main murine models employed to investigate the pathogenesis of celiac disease, highlighting their main biological features and experimental applications. HLA-DQ8 and HLA-DQ2.5 transgenic mice primarily model gluten-specific adaptive immune responses, whereas the NOD-DQ8-Ab^0^ model incorporates an autoimmune-prone background that enhances immune activation and barrier dysfunction. IL-15-driven HLA transgenic models represent the most pathophysiologically relevant systems, currently available reproducing gluten-dependent villous atrophy through the integration of innate and adaptive immunity. Non-transgenic mouse models are useful for investigating specific gluten-induced mechanisms and innate immune responses, although they do not reproduce the full spectrum of CeD pathology.

Nevertheless, reproducing the full spectrum of human CeD in mice remains challenging. Although several models successfully recapitulate key aspects of disease pathogenesis, including gluten-specific CD4^+^ T-cell responses and inflammatory cytokine production, spontaneous villous atrophy and the complete mucosal lesions characteristic of human disease are rarely observed in immunocompetent animals [116]. Consequently, individual murine models generally reflect specific components of CeD rather than the disease, making the complementary use of different systems necessary to address distinct pathogenic mechanisms. Pioneering studies were done in wild-type rodents and immunodeficient mouse strains, which provided the first experimental evidence linking gluten exposure to intestinal and immune alterations [117]. Currently, some non-transgenic murine models are still used, in which variable forms of gluten-dependent enteropathy can be experimentally induced. However, these systems are gradually being supplemented by increasingly complex transgenic mouse models that more closely resemble the characteristics of human CeD (Figure 3).

### 4.1. HLA-DQ8 Transgenic Mouse Models

The simplest HLA transgenic model used for CeD studies is the HLA-DQ8-Ab^0^ mouse. This model expresses the human HLA-DQ8 heterodimer (HLA-DQA103:01, DQB103:02) and lacks endogenous murine MHC class II, ensuring that CD4^+^ T-cell selection and activation depend on the human HLA transgene [118,119]. Gluten-specific immune responses are typically induced through immunization with gliadin or defined gluten peptides in the presence of adjuvants. Following sensitization, mice develop measurable HLA-DQ8-restricted CD4^+^ T-cell proliferation and cytokine secretion, particularly IFN-γ. Studies using this model have shown that gliadin immunization induces Th1-type cytokine production and recruitment of activated T cells to the small intestine, but does not generally induce villous atrophy, supporting the concept that adaptive immunity alone is insufficient for full mucosal damage observed in patients [120]. HLA-DQ8-Ab^0^ mice have also been used for the identification of immunodominant α-gliadin epitopes and for studying mechanisms of gluten-specific T-cell activation [121]. Although this model does not fully reproduce the epithelial destruction and intraepithelial lymphocytosis characteristic of active CeD, it can be used to study the intrinsic cytotoxicity and immune-related properties of wheat gliadin following specific sensitization protocols [120]. This model has also been used in translational studies. For instance, enzymatic transamidation of gliadin has been shown to reduce T-cell activation and IFN-γ production, supporting strategies aimed at reducing gluten immunogenicity [122,123].

The HLA-DQ8 transgenic mouse model has also been instrumental in investigating the impact of the microbiota on CeD pathogenesis, showing that microbial factors can influence the host response to gluten. Indeed, probiotic supplementation has been tested as a modulator of gliadin-driven immune responses [124]. In addition, microbiota composition has been shown to modulate the severity of gluten-induced immunopathology. Colonization with an enteroadherent *Escherichia coli* strain isolated from a patient with CeD abolished the protection observed in specific pathogen-free mice, while antibiotic-induced expansion of Proteobacteria exacerbated gluten-induced intestinal pathology, highlighting the contribution of microbial factors to disease development [125].

A mouse model more closely reflecting human physiology was obtained crossing HLA-DQ8-Ab^0^ transgenic mice with mice harboring a human CD4 transgene (hCD4/DQ8), in absence of endogenous murine MHC class II and CD4 genes [126]. When immunized with gluten, these mice also developed a strong antigen-specific T-cell proliferative response and anti-gliadin IgG antibodies but reproduced only specific immune features associated with CeD and did not develop the characteristic intestinal lesions observed in CeD patients. Early studies demonstrated that HLA-DQ8 promotes the recognition of both native and deamidated gluten peptides (DGPs), with the latter enhancing HLA-DQ8-restricted proliferative responses of CD4^+^ T cells.

Based on the evidence that adaptive immunity alone is insufficient to cause full mucosal damage, the combination of HLA-DQ8-Ab^0^ transgenic mice with the autoimmune-prone non-obese diabetic (NOD) genetic background (NOD-DQ8-Ab^0^ model) provides increased susceptibility to immune dysregulation and has been proposed as a system more permissive to gluten-induced mucosal alterations.

Indeed, following gliadin sensitization, these mice develop moderate enteropathy characterized by increased IELs and altered barrier function, resembling early Marsh I–II lesions rather than full villous atrophy [127]. Disease severity can be enhanced through experimental manipulation of immune regulatory pathways, including regulatory T-cell depletion [127]. Beyond intestinal pathology, the NOD-DQ8 background has also been exploited to model extraintestinal manifestations such as dermatitis herpetiformis-like lesions, supporting its relevance for gluten-associated autoimmunity [128]. This model has also served as a platform for testing antigen-specific tolerance strategies. Huibregtse et al. demonstrated that oral administration of *Lactococcus lactis* engineered to deliver a deamidated DQ8-restricted gliadin epitope can induce antigen-specific tolerance, suppress DQ8-restricted T-cell responses and increase Foxp3^+^ regulatory T cells [129].

Finally, an HLA-DQ8, hCD4 transgenic Ab0 NOD (hCD4/NOD-DQ8) mouse model was recently used in a preclinical study evaluating immunomodulatory nanoparticles (TIMP-GLIA), demonstrating that intravenous administration of TIMP-GLIA reduced gliadin-specific T-cell proliferation, inflammatory cytokine secretion, and circulating gliadin-specific IgG levels, while increasing FOXP3 expression and gene signatures associated with tolerance induction [130]. These findings support further investigation before progression to clinical trials in patients.

HLA-DQ8.Ab^0^ and hCD4/DQ8 transgenic mice provide a system for studying gluten-specific CD4^+^ T-cell immunity in vivo in a human-relevant HLA context. A major limitation is the frequent need for adjuvant-mediated immunization, which may not reflect natural dietary sensitization. Moreover, these models have limited potential for modeling the effector phase of tissue damage, as they do not develop full intestinal lesions.

Compared with HLA-DQ8 models, the NOD-DQ8-Ab^0^ system shows greater susceptibility to gluten-driven barrier dysfunction and immune activation. Nevertheless pathology remains moderate in this model as well. Additionally, autoimmune predisposition may introduce confounding inflammatory signals unrelated to CeD-specific mechanisms, limiting direct extrapolation to human disease. Finally, it is important to note that only a minority of patients with CeD are DQ8-positive whereas more than 95% carry DQ2-molecules. Although the genetic predisposing mechanisms associated with the two HLA molecules are similar—namely, high-affinity binding of deamidated gliadin peptides and their presentation to CD4^+^ T cells—the variety and number of DQ2-restricted epitopes are much greater than those of DQ8-restricted epitopes.

### 4.2. HLA-DQ2.5 Transgenic Mouse Models

Based on these considerations, HLA-DQ2.5 transgenic mouse models were generated to better reflect the genetic profile of most patients with CeD. de Kauwe et al. [131]. generated HLA-DQ2/CD4 transgenic mice lacking murine MHC class II molecules and expressing human CD4 and the HLA-DR3-DQ2 haplotype (hCD4/DR3-DQ2). The authors demonstrated that, although this model developed HLA-DQ2-restricted, gliadin-specific CD4^+^ T-cell responses following immunization with immunodominant gliadin epitopes, neither dietary gluten exposure nor pre-existing gluten-specific immunity alone were sufficient to induce intestinal pathology. Only the combination of gluten exposure and intestinal inflammation resulted in low-penetrance enteropathy and tissue transglutaminase-specific IgA production [131]. This transgenic mouse model confirms observations derived from HLA-DQ8 models, indicating that gluten ingestion can be tolerated without intestinal pathology even when HLA-DQ2-restricted CD4^+^ T-cell immunity to gluten is established. Recently, this model was used to investigate IEC-gluten-Tcell interactions through co-cultures of organoid-derived monolayers with CD4^+^ T cells from immunized mice [132].

A second model was developed by Du Pré aand colleagues. This model combines expression of the human HLA-DR3-DQ2 haplotype with a transgenic gliadin-specific T-cell receptor recognizing an HLA-DQ2-restricted gluten epitope from α gliadin (DR3-DQ2/gliaTCR). It enabled the comparison of gluten-specific T-cell responses with tolerogenic mucosal T-cell responses to ovalbumin, which was selected as a model food protein. The findings suggest that, under homeostatic conditions, deamidated gliadin induces a tolerogenic type 1 regulatory T-cell response rather than pathogenic immunity, with tolerance being maintained through IL-10-dependent mechanisms in the spleen [133]. Lately, a TCR transgenic mouse with CD4^+^ T cells recognizing a different immunodominant peptide derived from ω-gliadins (DQ2.5-glia-ω2 gluten epitope) was generated and characterized [134].

Finally, an HLA-DQ2.5 knock-in mouse strain on a C57BL/6 background was generated to avoid the need for crossing with endogenous MHC class II knockout mice, and to facilitate the study of the isolated function of the HLA transgene [135].

From a translational perspective, these models have the potential to serve as preclinical platform for antigen-specific immunotherapies and tolerance-inducing strategies targeting HLA-DQ2-restricted epitopes. Indeed, HLA-DQ2.5 transgenic mice lacking endogenous murine MHC class II molecules (HLA-DQ2.Ab^0^), have been used to evaluate highly selective immunotherapeutic approaches targeting pathogenic HLA-DQ2.5–gluten peptide complexes. Using this platform, Igawa et al. demonstrated that the monoclonal antibody DONQ52 selectively inhibited gluten-specific T-cell activation while preserving immune responses against unrelated antigens, highlighting the value of HLA-DQ2-based models for preclinical assessment of antigen-specific therapies [136].

Overall, these models are particularly useful for mechanistic investigation of T-cell priming, cytokine profiles, and trafficking in vivo. However, they reproduce only selected immune features of CeD and do not fully reproduce villous atrophy, intraepithelial lymphocytosis, or the complete spectrum of intestinal pathology. Moreover, the use of a single dominant TCR limits the representation of the polyclonal T-cell repertoire present in patients.

### 4.3. IL-15-Driven Mouse Models

IL-15 is a central cytokine in CeD pathogenesis, promoting IELs activation and licensing cytotoxic epithelial damage. To investigate the synergy between adaptive gluten-specific immunity and innate epithelial stress, IL-15-driven transgenic mouse models have been developed. Among transgenic models, T3(b)-hIL-15 transgenic mice, which overexpress human IL-15 in enterocytes, recapitulate several key features of CeD, including increased number of CD8^+^ T cells in the small intestine, development of anti-TG2 autoantibodies, and marked plasmacytosis in lamina propria [137]. The same research group subsequently used this transgenic model to assess the therapeutic effects of a pan-JAK inhibitor that abrogates IL-15 signaling in T3(b)-hIL-15 transgenic mice [138].

When these mice were crossed with mice carrying ovalbumin-specific CD4^+^ T cells, the combination of IL-15 expression and CD4^+^ T cells was sufficient to activate CD8^+^ T cells and damage the small intestine in mice fed the relevant food antigen [117].

In another model, IL-15 overexpressing mice were crossed with DQ8 transgenic mice. The resulting IL-15/DQ8 transgenic mice developed increased number of CD3^+^ T cells and IELs in response to gliadin feeding [139].

Finally, Abadie et al. [140] developed the DQ8-Dd-villin-IL-15 transgenic mouse model, integrating three key disease components: HLA-DQ8 expression, gluten exposure, and IL-15 overexpression in epithelial and hematopoietic compartments driven by the villin promoter [141]. Upon dietary gluten exposure, these mice develop gluten-dependent villous atrophy and inflammatory immune activation without the need for exogenous adjuvants. Importantly, key histopathological features of CeD develop only when IL-15 is expressed in both epithelial and hematopoietic cells, highlighting the compartment-specific contribution of IL-15 signaling [140].

This system has enabled the mechanistic dissection of interactions between gluten-specific CD4^+^ T cells and cytotoxic IEL responses. It has also supported investigation of TG2 activation and gluten deamidation in vivo, reinforcing the physiological relevance of antigen modification mechanisms [140,141].

DQ8-Dd-villin-IL-15 is among the most pathophysiological relevant murine systems currently available for studying key features of CeD. Its main limitations are the complexity of the required genetic engineering and the high technical demands of its use. In addition, IL-15 overexpression is an artificial driver that may exceed physiological levels observed in patients. Moreover, the model does not fully reproduce the diversity of human genetic backgrounds, microbiota influences, and environmental modifiers.

### 4.4. Non-Transgenic Mouse Models

In addition to transgenic models, several studies have employed non-transgenic mouse strains to investigate gluten-induced intestinal pathology and possible immunomodulatory strategies. In these models, enteropathy can be experimentally induced through gluten sensitization, adoptive transfer of gliadin-reactive lymphocytes, or administration of immunogenic gliadin peptides.

For example, Freitag et al. [142] demonstrated that adoptive transfer of gliadin-primed CD4^+^CD45RB^lowCD25^−^ T cells into lymphogenic mice induced gluten-dependent small-intestinal damage. Similarly, Araya et al. [143] showed that intraluminal administration of the immunogenic gliadin peptide p31–43 in C57BL/6 mice triggered intestinal inflammation and epithelial cell death through MyD88- and type I interferon-dependent pathways. In another model, prolonged oral and intraperitoneal administration of gliadin to BALB/c mice induced gluten-associated inflammation accompanied by IL-15-mediated immune activation [144]. These models are particularly suitable for investigating the toxic effects of gluten proteins, the induction of innate immune responses and mechanisms involved in the breakdown of oral tolerance. However, they reproduce only some aspects of CeD pathogenesis and are therefore less suitable for investigating the HLA-restricted adaptive immune responses that characterize the disease.

## 5. Contributions of Experimental Models to the Development of Therapies for Celiac Disease

The preclinical evidence supporting the main therapeutic strategies that have reached the clinical trial phase, are summarized in Table 1. Clinical results are not yet available for many of the compounds listed, and some have not been tested in the study models discussed in this review. Nevertheless, for selected therapeutic classes—including glutenases, intestinal permeability modulators, anti-IL-15 antibodies, and immuno-modulatory agents—it is possible to compare preclinical findings with results from studies in patients with CeD.

It is evident that not all compounds were developed using disease-specific celiac models; rather, the extent to which preclinical testing included celiac-relevant cell systems, organ cultures, or animal models varies substantially across therapeutic classes.

For therapies intended to detoxify gluten by enhancing its gastric digestion, including latiglutenase (ALV003), AN-PEP, TAK-062 and STAN1, gluten-reactive T-cell lines and T-cell clones have been largely used [35,36,37,39]. Early studies of the enzyme combination underlying latiglutenase showed that EP-B2 and SC PEP acted synergistically to reduce gluten immunotoxicity under simulated gastric conditions [35]. However, digested whole-wheat bread could still elicit T-cell responses at high protein doses, indicating incomplete detoxification. Clinical findings have been inconsistent: some trials reported reductions in gluten-induced mucosal injury or symptom severity, whereas others found no significant benefit over placebo [145]. A 2025 meta-analysis likewise found no significant overall improvement in histological outcomes [145]. Similarly, Stepniak et al. [36] showed that AN-PEP degraded all the tested T-cell-stimulatory peptides as well as intact gluten molecules, in vitro. However, an exploratory randomized controlled trial in patients with CeD following a long-term gluten-free diet found no significant reduction in overall stool gluten-immunogenic-peptide concentrations or changes in CeD-specific serology [145]. Overall, although in vitro T-cell assays alone have limited ability to predict clinical efficacy on its own, residual T-cell activation may serve as an indicator of incomplete gluten detoxification.

Agents targeting intestinal barrier dysfunction, mainly larazotide acetate (AT-1001), have been evaluated in a broader range of disease-relevant systems, including intestinal epithelial cell lines [147], human intestinal organ cultures [71], and hCD4/DQ8 transgenic mice [147]. Drago et al. [71] showed that a zonulin antagonist could prevent gliadin-induced tight-junction disruption and increased paracellular permeability in epithelial cells and human intestinal biopsies. Subsequent studies using epithelial cell lines showed that larazotide preserved barrier integrity and reduced the passage of immunogenic gliadin peptides across cell monolayers [147]. In mice, larazotide treatment also preserved tight-junction structure and reduced intestinal macrophage accumulation [147]. Larazotide acetate was subsequently evaluated in four phase 2 clinical trials [145]. A meta-analysis of randomized controlled trials reported reductions in the frequency and severity of gastrointestinal symptoms during gluten challenge, but found no significant improvement in intestinal permeability [145]. Overall, the preclinical studies show that larazotide can protect the epithelial barrier in experimental models. However, these effects have not consistently translated into measurable improvements in intestinal permeability or clinical outcomes.

Preclinical approaches for other non-immunomodulatory strategies have been more heterogeneous. The gluten-sequestering compound BL-7010 was studied in epithelial cell lines [69] and in two murine models of gluten sensitivity, hCD4/DQ8 [69], and NOD-DQ8-Ab^0^ mice [146]. By contrast, AGY-010 was evaluated primarily in conventional murine feeding studies rather than dedicated CeD models [151]. ZED1227, a TG2 inhibitor, has been investigated using Caco-2 cells and patient-derived intestinal organoids [72,111].

Anti-inflammatory strategies have also used different experimental systems, according to their molecular targets. DONQ52, a T-cell receptor–blocking antibody, was evaluated in HLA-DQ2.5 transgenic mice [136]. Drugs targeting IL-15, such as CALY-002 and PRV-015, were supported by studies in IL-15 transgenic mice [149,150]. PRV-015 was also examined in organ cultures derived from patients with refractory celiac disease [150] and in rhesus macaques [159]. Work by Malamut et al. using human tissue cultures and IL-15-transgenic mice identified a plausible mechanism through which IL-15 promotes intraepithelial lymphocyte survival, involving the IL-15–IL-15Rβ–JAK3–STAT5 pathway, and showed that neutralizing IL-15 could interrupt this process in human tissue [150]. However, trials of AMG 714 in patients with refractory CeD did not demonstrate convincing improvements in the principal histological outcomes, despite some symptomatic improvement [145].

Several other compounds currently being explored for CeD originated in therapeutic programs developed for inflammatory bowel disease, autoimmune disorders, or immune-cell signaling models rather than dedicated CeD models. These include PTG-100, ritlecitinib, tofacitinib, IMU-856, vedolizumab, vercirnon and amlitelimab [148,152,153,154,155,156,157]. For example, PTG-100 was evaluated in DSS-induced colitis [153], tofacitinib in T84 intestinal epithelial cells [155] and IL-15-overexpressing mice [138], while ritlecitinib was studied in experimental autoimmune encephalomyelitis and alopecia areata models [156]. Among these candidates, tofacitinib provides an example of concordant evidence across experimental and clinical settings, particularly in refractory CeD type II (RCDII). In vitro studies showed that tofacitinib, through inhibition of JAK–STAT signaling, protected intestinal epithelial cells against cytokine-induced barrier dysfunction by preserving tight-junction organization, reducing permeability, and restoring barrier function after inflammatory damage. In IL-15-transgenic mice, treatment reduced intestinal inflammation and duodenal pathology, alongside a marked reduction in pathogenic CD8^+^ T lymphocytes [138].

Clinical findings in patients with RCDII were consistent with several of these preclinical observations [145]. Tofacitinib was associated with rapid improvement in gastrointestinal symptoms and weight gain, while histological analyses showed recovery of villous architecture in most patients. However, the proportion of aberrant IELs remained largely unchanged. Unlike the reduction in CD8+/ aberrant IEL populations observed in mice, the human findings suggest that tofacitinib may primarily suppress the pathogenic activity of the abnormal lymphocytes rather than eliminate the population itself.

Antigen-specific tolerization strategies show variable degrees of CeD-specific preclinical validation. NexVax2 entered human clinical testing without reported in vitro or in vivo model studies. TPM502 (derived from Topas Particle Conjugate), was supported mainly by tolerance-induction platforms developed in non-CeD autoimmune models, including type 1 diabetes and experimental autoimmune encephalomyelitis [158], while no preclinical model studies are available for KAN-101 [160]. In contrast, TIMP-glia/TAK-101 was evaluated in several mouse models, including HLA-DQ8 and human CD4 transgenic NOD mice [142], thus providing direct evidence in a gliadin-responsive system. Freitag et al. reported that intravenous administration of these gliadin-loaded nanoparticles induced antigen-specific immune tolerance in mouse models [142]. Treatment reduced gliadin-specific T-cell proliferation, inflammatory cytokine production, antibody responses, and intestinal damage, and increased markers associated with immune tolerance, including FOXP3 expression. Subsequently, in a randomized, double-blind, placebo-controlled phase 2a trial involving 33 patients, Kelly et al. [161] found that intravenous TAK-101 reduced circulating gliadin-specific IFN-γ-producing T cells after oral gluten challenge. Treatment also appeared to limit deterioration in the villous-to-crypt ratio, although this difference was not statistically significant. Although the treatment was generally well-tolerated, with no serious adverse events reported, there were no significant improvements in clinical symptoms or IEL percentages [161]. The TAK-101 trial therefore provided partial clinical confirmation of the preclinical findings, particularly for the reduction of gluten-specific inflammatory T-cell responses. However, the regulatory T-cell mechanisms observed in mice were not conclusively demonstrated in humans, and effects on intestinal histology and symptoms remain unconfirmed.

Overall, to date, the evidence from comparison between preclinical studies and clinical trials caution against predicting therapeutic success from in vitro pathway inhibition or experimental models alone. Nevertheless, a likely explanation is that many of the models used so far only capture selected aspects of CeD and therefore do not adequately reflect its biological complexity. Importantly, emerging models that more closely recapitulate tissue organization and disease pathophysiology have not yet been extensively incorporated into drug evaluation pipelines. The closer agreement between preclinical and clinical findings reported for TAK-101 and tofacitinib in selected mouse models supports the notion that model choice may substantially influence translational predictability. Consequently, advanced human-relevant platforms may improve the assessment of novel therapies, although their predictive performance still requires rigorous validation.

## 6. Discussion and Future Perspectives

Overall, the diverse models currently available provide complementary insights into distinct biological mechanisms underlying CeD, each offering specific strengths, while presenting inherent limitations. Intestinal T-cell lines and epithelial cell models enable the controlled dissection of antigen-specific immune responses and early epithelial events, respectively, but remain reductionist systems that lack the structural complexity and multicellular interactions found in vivo. Ex vivo intestinal organ cultures preserve native tissue architecture and provide a direct link between molecular events and mucosal injury, although their limited viability restricts long-term studies. Patient-derived organoids offer physiologically relevant and potentially patient-specific platforms for studying epithelial responses and persistent disease-associated features, but their predominantly epithelial composition remains a limitation. ALI organoids and immune–epithelial co-culture systems address this limitation by incorporating additional cell types and enabling epithelial–immune interactions, although they remain technically demanding, variable and in need of validation across multiple laboratories. Organ-on-chip can integrate epithelial, immune, microbial, and environmental factors under dynamic conditions, yet their application to CeD remains underdeveloped.

Finally, murine models enable investigations that are not feasible in other systems, including systemic immune priming, lymphocyte trafficking, tolerance, microbiota-dependent effects and long-term disease progression. Among these, non-transgenic mouse models are useful for investigating selected immune and gluten-related effects but have limited disease specificity. HLA-DQ2/DQ8 transgenic mice are particularly useful for studying gluten-specific antigen presentation and adaptive immunity, but do not fully reproduce intestinal pathology. IL-15-driven models, including more advanced models combining IL-15 overexpression with an HLA-DQ8 background, capture immune–epithelial interactions and can produce villus atrophy. Nevertheless, even these models should not be interpreted as a definitive representation of human CeD, because their dependence on supraphysiological IL-15 expression and engineered genetics background may amplify particular pathways at the expense of others.

It is important to note that, because individual systems capture distinct components of CeD pathogenesis, the inability of a model to reproduce a specific phenotype may reveal a biological component or cellular interaction that is missing from that system. Accordingly, these models should be considered complementary tools that can be used sequentially or in parallel. Findings obtained using basic cellular systems can be evaluated on more complex models; conversely, observations made in vivo can be investigated in greater detail using ex vivo and in vitro systems. Future studies should therefore focus not only on refining individual models but also on combining models across different levels of biological complexity to obtain complementary information. Increasing attention should also be paid to the role of the gut microbiota in CeD pathogenesis. Growing evidence suggests that alterations in the intestinal microbiota may influence gluten-driven immune responses and contribute to the variability of disease manifestations among patients [162]. Therefore, future experimental systems should aim to incorporate microbiota-host interactions, helping to clarify how environmental factors influence the transition from gluten tolerance to chronic intestinal inflammation. Such integrated approaches could help address unresolved questions concerning non-HLA genetic susceptibility, the contribution of microbiota, gluten trafficking and antigen presentation, loss of oral tolerance, the roles of regulatory and unconventional lymphocyte populations, and the sequence of events linking innate and adaptive immune activation to epithelial injury and tissue remodeling.

In this context, emerging technologies offer new opportunities to address gaps in our understanding of CeD pathogenesis and improve the translational relevance of research. CRISPR-based genome editing can be used to establish causal relationships between genetic variants and disease-associated cellular phenotypes, enabling the targeted investigation of non-HLA susceptibility genes and signaling pathways, including those driven by IL-15 and the newly identified role of IL-7. Single-cell transcriptomic could help resolve disease-associated epithelial, stromal, myeloid, and lymphocyte states and, potentially elucidate the temporal sequence of cellular responses to gluten. Spatial multiomics approaches could be used to map these cell populations, characterize their molecular states, and determine their organization within affected tissues. Advanced imaging technologies, including live-cell microscopy and multiplexed imaging, may further enable researchers to link the molecular characteristics of cells to their behavior and investigate processes such as immune-cell migration, cellular interactions, cell killing, epithelial cell loss, and barrier disruption.

When integrated with organoids, co-culture systems, organ-on-chip technologies, and humanized mouse models, these powerful approaches have the potential to deepen our understanding of how genetic susceptibility, environmental triggers, and multicellular responses converge to drive disease development.

## 7. Conclusions

No single experimental model fully recapitulates the complexity of CeD, which arises from the interplay of epithelial, immune, stromal and microbial components, in addition to genetic and environmental factors.

Nevertheless, the use of in vitro, ex vivo, and in vivo systems has provided valuable tools for investigating disease mechanisms, substantially advancing our understanding of CeD pathogenesis and supporting the development of new therapeutic agents. Despite these advances, several key aspects of CeD pathogenesis remain unclear. At the same time, several therapeutic agents have advanced to clinical testing without extensive validation in disease-specific preclinical systems, underscoring the continuing need for physiologically relevant and accessible experimental models.

Currently available models and next generation experimental systems are expected to play a pivotal role in addressing these challenges. Patient-derived epithelial organoids, ALI organoids and emerging organ-on-chip technologies offer new opportunities to investigate epithelial–immune–microbial interactions in a human-relevant context.

At the same time, increasingly sophisticated genetically engineered mouse models and IL-15-driven systems provide powerful tools to study disease initiation, tolerance breakdown, long-term immune responses and mucosal damage progression in vivo.

The future of CeD modeling is unlikely to depend on a single optimized system. Instead, progress will depend on the integration of complementary experimental models with technologies that enable the perturbation, characterization, and visualization of disease processes at cellular and spatial resolution. Ultimately, this integration could help address outstanding questions in disease pathogenesis while accelerating the identification and supporting the validation of new therapeutic targets and innovative strategies aimed not only at controlling inflammation but also at restoring durable immune tolerance to gluten.

## Figures and Tables

**Table 1:** Clinical trial identifiers refer to the latest registered studies evaluating the safety and efficacy of each agent. Preclinical models include celiac disease-specific systems (e.g., HLA-DQ2/DQ8 transgenic mice, IL-15 models, intestinal organ cultures, and gluten-specific T-cell lines) as well as general experimental models used for mechanistic studies. “No specific models of celiac disease” indicates the absence of a validated CeD-specific preclinical system.

Agent Name	Molecule/Action	Clinical Trial	Preclinical Study Model (REF)
Latiglutenase/ALV003	EP-B2 + SC-PEP enzymes/ Gluten degradation	ClinicalTrials.gov ID: NCT04839575	T-cell lines [35]
AN-PEP/Tolerase G	AN-Prolyl endopeptidase/ Gluten degradation	ClinicalTrials.gov ID: NCT04788797	T-cell lines [36]
TAK062/Kuma062/	Engineered glutenase/ Gluten degradation	ClinicalTrials.gov ID: NCT05353985	T-cell lines [39]
STAN1	DPP-IV+ Aspergillopepsin/ Gluten degradation	ClinicalTrials.gov ID: NCT00962182	T-cell lines [37]
Larazotide Acetate/AT-1001	Tight-junction modulator; Syntetic peptide/reducing intestinal permeability	ClinicalTrials.gov ID: NCT03569007	Caco2 and IEC6 cells [145] Organ culture [71] hCD4/DQ8 transgenic mouse [145]
AGY-010	Anti-gliadin antibody/ Gluten sequestration	US Clinical Trial NCT03707730	No specific models of CeD [146]
BL-7010	Synthetic polymer/ Gluten sequestration	ClinicalTrials.gov ID: NCT01990885	IEC-6, Caco2/15 [69] hCD4/DQ8 transgenic mouse [69] NOD-DQ8 mouse model [147]
IMU-856	Small molecule targeting SIRT6/ Immune modulation	Australian New Zealand Registry ACTRN12620000901909	Caco-2 cells [148] No specific mouse models of CeD [148]
ZED1227/Tak-227	Small molecule TG2 inhibitor/ Immune modulation	ClinicalTrials.gov ID NCT05818956	Caco-2 cells [72] PDOs generated after in vivo trial [111]
DONQ52	moAb targeting HLA-DQ2.5–gluten peptide complex/ Immune modulation	ClinicalTrials.gov NCT07239336	HLA-DQ2.5 transgenic mice [136]
PRV-015 (former AMG 714)	Anti-IL-15 moAb/ Immune modulation	ClinicalTrials.gov NCT04424927	Organ culture of RCD patients [149] IL-15 transgenic mouse [149] GS Rhesus Macaques [150]
Caly-002	Anti-IL-15 moAb/ immune modulation	ClinicalTrials.gov NCT04593251	IL-15 transgenic mouse [151]
Vedolizumab	Anti-a4b7 integrin moAb/ Immune modulation	ClinicalTrials.gov NCT02929316	No specific models of CeD [152]
PTG-100	Synthetic peptide targeting a4b7 integrin/ Immune modulation	ClinicalTrials.gov NCT04524221	No specific models of CeD [153]
GSK1605786A/Vercirnon (Formerly CCX282-B)	Small molecule CCR9 antagonist/ Immune modulation	ClinicalTrials.gov NCT00540657	No specific models of CeD [154]
Tofacitinib	Small molecule JAK inhibitor (JAK1/JAK3); Immune modulation	EudraCT 2018-001678-10	T84 cell lines [155] T3(b)-hIL-15 Tg mouse [138]
Ritlecitinib/ATI-2138	Small molecule JAK inhibitor/(selective JAK3/TEC family) Immune modulation	ClinicalTrials.gov NCT05636293	No specific models of celiac disease [156]
Amlitelimab	moAb anti-OX40L/ Immune modulation	ClinicalTrials.gov NCT06557772	No specific models of celiac disease [157]
NexVax2	Synthetic gluten-derived peptides/ Gluten Tolerization	ClinicalTrials.gov NCT03644069	No specific models of celiac disease
TIMP-glia/TAK101	Tolerogenic. Nanoparticles/ Gluten tolerization	ClinicalTrials.gov NCT04530123	huCD4/DQ8 transgenic Ab0 NOD mice [130]
KAN-101	Tolerogenic Nanoparticles/ Gluten tolerization	ClinicalTrials.gov NCT06001177	No specific models of CeD
TPM502	Tolerogenic nanoparticles/ Gluten tolerization	ClinicalTrials.gov NCT05660109	No specific models of CeD [158]

## Data Availability

No new data were created or analyzed in this study. Data sharing is not applicable to this article.

## Abbreviations

The following abbreviations are used in this manuscript:

Abbreviation	Definition
ALI	Air–liquid interface
AN-PEP	*Aspergillus niger* prolyl endopeptidase
APC	Antigen-presenting cell
ATIs	Amylase–trypsin inhibitors
CeD	Celiac disease
CFTR	Cystic fibrosis transmembrane conductance regulator
DGP	Deamidated gliadin peptide
DPP-IV	Dipeptidyl peptidase IV
EGF	Epidermal growth factor
EGFR	Epidermal growth factor receptor
EMA	Anti-endomysial antibodies
EP-B2	Barley cysteine endoprotease B2
ER	Endoplasmic reticulum
FOXP3	Forkhead box P3
GFD	Gluten-free diet
GEVs	Gut-Ex Vivo System
IELs	Intraepithelial lymphocytes
IEC	Intestinal epithelial cell
ILCs	Innate lymphoid cells
iPSC	Induced pluripotent stem cell
iTCLs	Intestinal T-cell lines
JAK	Janus kinase
MICA/B	MHC class I polypeptide-related sequence A/B
moAb	monoclonal Antibody
mTOR	Mechanistic target of rapamycin
MyD88	Myeloid differentiation primary response 88
NF-κB	Nuclear factor kappa B
NKG2D/NKG2C	Natural killer group 2 member D/C receptors
NOD	Non-obese diabetic
PBMCs	Peripheral blood mononuclear cells
PDOs	Patient-derived organoids
PDMS	Polydimethylsiloxane
PTPRK	Protein tyrosine phosphatase receptor type K
RCD	Refractory celiac disease
ROS	Reactive oxygen species
SIRT6	Sirtuin 6
TEER	Transepithelial electrical resistance
TG2	Tissue transglutaminase 2
TIMP-GLIA	Tolerogenic immune-modulating particle targeting gliadin
TNF-α	Tumor necrosis factor alpha
ZO-1	Zonula occludens-1
